# Robotic total knee arthroplasty with functional positioning safely addresses major coronal deformities: Comparable complications and survivorship

**DOI:** 10.1002/jeo2.70613

**Published:** 2026-01-12

**Authors:** Luca Andriollo, Emanuele Diquattro, Christos Koutserimpas, Giovan Giuseppe Mazzella, Giulio Bonat, Elvire Servien, Cécile Batailler, Sébastien Lustig

**Affiliations:** ^1^ Orthopaedics Surgery and Sports Medicine Department FIFA Medical Center of Excellence, Croix‐Rousse Hospital, Lyon University Hospital, Hospices Civils de Lyon Lyon France; ^2^ Ortopedia e Traumatologia, Fondazione Poliambulanza Istituto Ospedaliero Brescia Italy; ^3^ Artificial Intelligence Center, Alma Mater Europaea University Vienna Austria; ^4^ SC Ortopedia‐Traumatologia e Chirurgia Protesica e dei Reimpianti di Anca e Ginocchio, IRCCS Istituto Ortopedico Rizzoli Bologna Italy; ^5^ 2^nd^ Department of Orthopaedic Surgery ‘Hygeia’ General Hospital of Athens Athens Greece; ^6^ School of Rehabilitation Health Sciences, University of Patras Patras Greece; ^7^ Department of Aging Orthopaedic and Rheumatological Sciences, Fondazione Policlinico Universitario Agostino Gemelli IRCCS Rome Italy; ^8^ LIBM‐EA 7424, Interuniversity Laboratory of Biology of Mobility, Claude Bernard Lyon 1 University Lyon France; ^9^ Univ Lyon, Claude Bernard Lyon 1 University, IFSTTAR, LBMC UMR_T9406 Lyon France

**Keywords:** functional alignment, functional knee positioning, major deformities, TKA, valgus, varus

## Abstract

**Purpose:**

Robotic‐assisted total knee arthroplasty (TKA) has emerged as a reliable strategy to improve surgical accuracy and enable functional alignment (FA), also referred to as functional knee positioning (FKP). However, its application in patients with major coronal plane deformities remains under‐investigated. This study aimed to evaluate complication rates, implant survival, radiographic outcomes and patient‐reported measures in patients with severe deformities undergoing robotic‐assisted TKA with FA/FKP principles compared to matched controls.

**Methods:**

A retrospective comparative study was conducted on patients who underwent robotic‐assisted TKA between March 2021 and February 2023 at a single high‐volume centre. Patients with ≥15° varus or ≥10° valgus deformity were included in the study group and matched 1:1 with controls presenting neutral alignment. All procedures used the Mako robotic‐arm‐assisted system with standardised FA/FKP principles. Clinical outcomes included knee society score (KSS), forgotten joint score (FJS‐12), Kujala anterior knee pain scale (AKPS) and range of motion. Radiographic measurements and robotic data were assessed. Complications, reoperations and revision rates were analysed.

**Results:**

Eighty‐eight patients (44 per group) were analysed, with a mean follow‐up of 2.8 ± 0.9 years. Complication and revision rates were comparable between groups (revision: 2.3% vs. 0%, *p* = 0.987). Patients with major deformities achieved higher FJS‐12 scores (83.9 ± 20.2 vs. 74.9 ± 19.0, *p* = 0.040), although the difference did not exceed the minimal clinically important difference (MCID = 9.9). Postoperative mHKA was less neutral in the deformity group (176.8° ± 4.7 vs. 180.0° ± 3.0, *p* = 0.002), without adverse impact on implant survival.

**Conclusions:**

Robotic‐assisted TKA performed with FA/FKP principles appears to be a feasible option for patients with severe varus or valgus deformities. Despite residual alignment variability, complication and revision rates remained comparable to standard cases, and patient‐reported outcomes suggested greater perceived functional improvement.

**Level of Evidence:**

Level III.

## Graphical abstract

Robotic‐assisted TKA performed with functional knee positioning shows comparable short‐term safety and functional outcomes in patients with major coronal deformities.

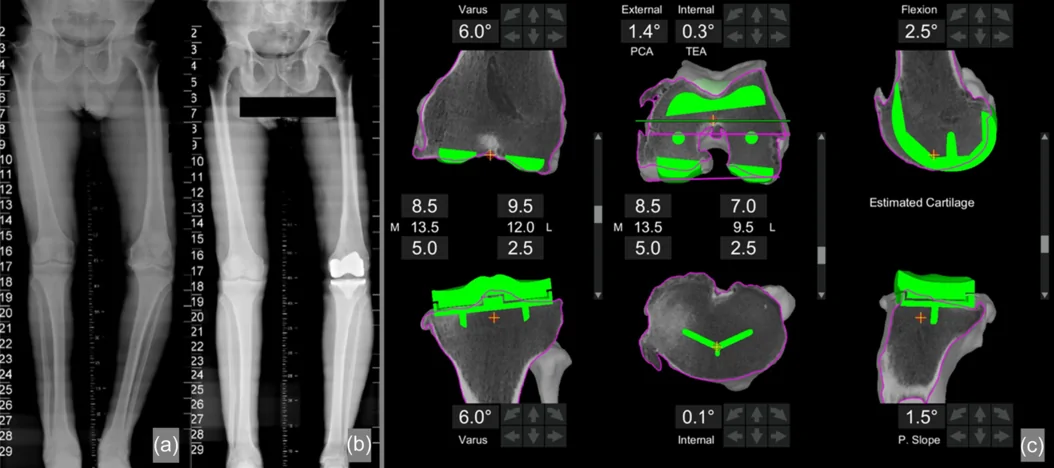

**Abbreviations**
AKPS: Kujala Anterior Knee Pain Scale
CS: cruciate substituting
DAIR: debridement, antibiotics and implant retention
FA: functional alignment
FJS‐12: Forgotten Joint Score‐12
FKP: functional knee positioning
ICC: intraclass correlation coefficient
KSS: knee society score
LDFA: lateral distal femoral angle
MCID: minimal clinically important difference
mHKA: mechanical hip‐knee‐ankle angle
MPTA: medial proximal tibial angle
MUA: manipulation under anaesthesia
OKS: Oxford knee score
PROMs: patient‐reported outcome measures
PS: posterior stabilised
ROM: range of motion
sTEA: surgical transepicondylar axis
TKA: total knee arthroplasty
TS: tibial slope

## INTRODUCTION

Robotic‐assisted total knee arthroplasty (TKA) has shown promising results in recent years, particularly in enhancing surgical accuracy and patient‐reported outcome measures (PROMs) [8, 14, 28].

With robotic technology, it is now possible to pursue individualised or functional alignment (FA) strategies that account for three‐dimensional anatomy and soft tissue balance [1, 2, 3, 19]. Ensuring correct coronal alignment is essential for both functional improvement and long‐term implant survivorship [18, 20, 27].

Traditionally, severe varus or valgus deformities have been considered challenging in TKA, often leading to increased technical complexity, higher risk of residual imbalance, and the potential need for constrained implants [4, 7, 23, 26]. The standard goal has been to restore a neutral mechanical axis, but this approach may not always reflect patient‐specific anatomy [13, 29].

Robotic assistance provides surgeons with the ability to execute precise bone resections and tailor implant positioning, potentially reducing the need for extensive soft tissue release and offering more reproducible outcomes, even in anatomically complex knees [1, 2, 3]. Recent studies have demonstrated the feasibility of FA in robotic TKA. However, evidence specifically addressing cases with major deformities (≥15° varus or ≥10° valgus) remains limited [27].

In this study, patients with major coronal deformities were compared with those with standard alignment undergoing robotic‐assisted TKA performed with FA principles, also referred to as functional knee positioning (FKP). The primary endpoint was the comparison of PROMs between the two groups. Secondary endpoints included complication rates, implant survival and radiographic parameters. It was hypothesised that robotic TKA would achieve comparable safety profiles in complex deformities while potentially improving joint perception and functional recovery.

## METHODS

This retrospective comparative study utilised a prospectively maintained database of patients who underwent robotic‐assisted TKA between March 2021 and February 2023. Patients were divided into two groups based on the presence or absence of major coronal plane deformities.

The study group included patients with a coronal plane deformity of ≥15° varus or ≥10° valgus, while the control group comprised patients with a mechanical hip‐knee‐ankle (mHKA) angle ranging between 166° and 179°. Preoperative coronal plane deformities were assessed using full‐length standing X‐rays.

**Figure 1:**
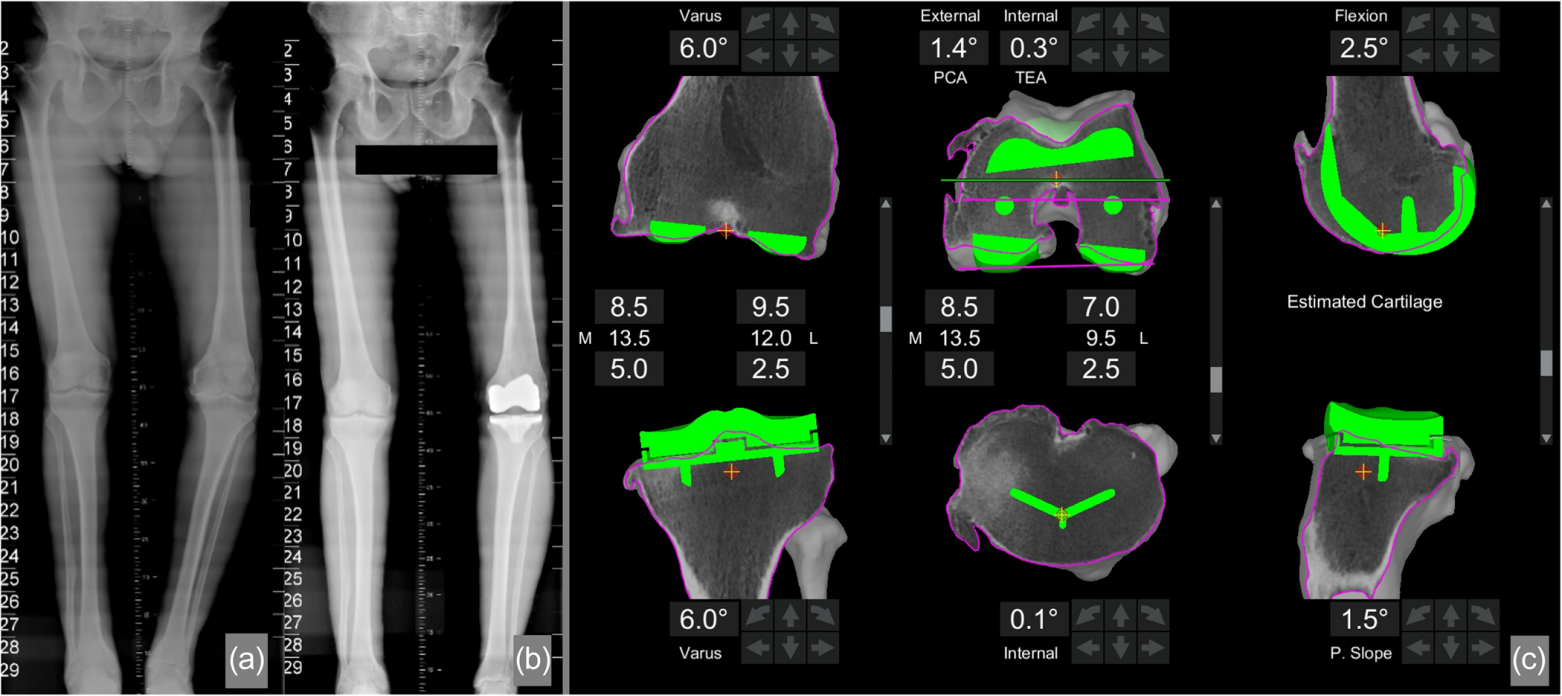
Seventy‐six‐year‐old female patient with a major varus coronal deformity, presenting a preoperative mechanical hip–knee–ankle (mHKA) angle of 161° on the left side, corrected to 167° after total knee arthroplasty (TKA). (a) Preoperative long‐leg standing radiograph; (b) postoperative long‐leg standing radiograph; (c) intraoperative screenshot of TKA performed with the Mako® robotic system (Stryker ®), showing planning adjustments according to functional knee positioning principles.

**Figure 2:**
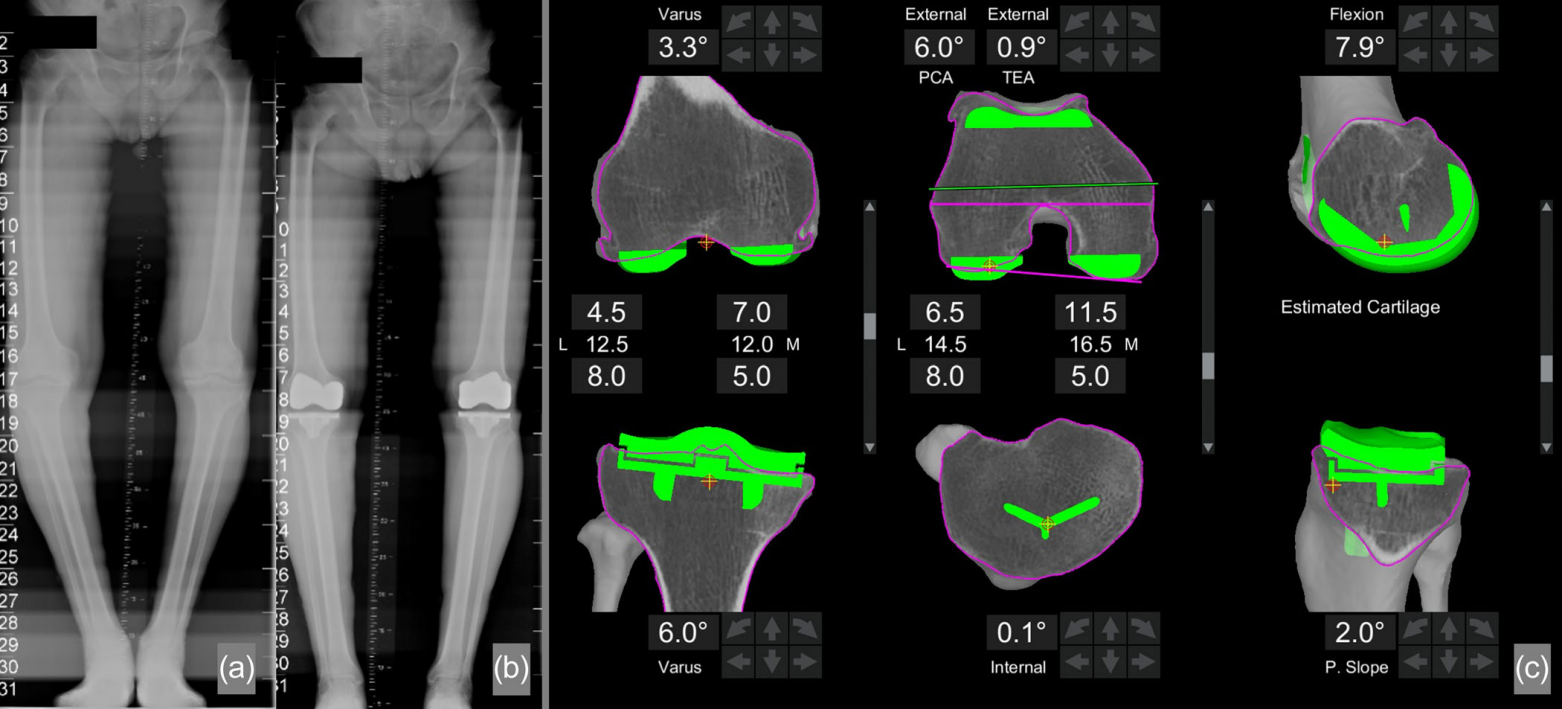
Seventy‐five‐year‐old male patient with a major varus coronal deformity, presenting a preoperative mechanical hip–knee–ankle (mHKA) angle of 161° on the right side, corrected to 171° after total knee arthroplasty (TKA). (a) Preoperative long‐leg standing radiograph; (b) postoperative long‐leg standing radiograph; (c) Intraoperative screenshot of TKA performed with the Mako® robotic system (Stryker ®), showing planning adjustments according to functional knee positioning principles.

**Figure 3:**
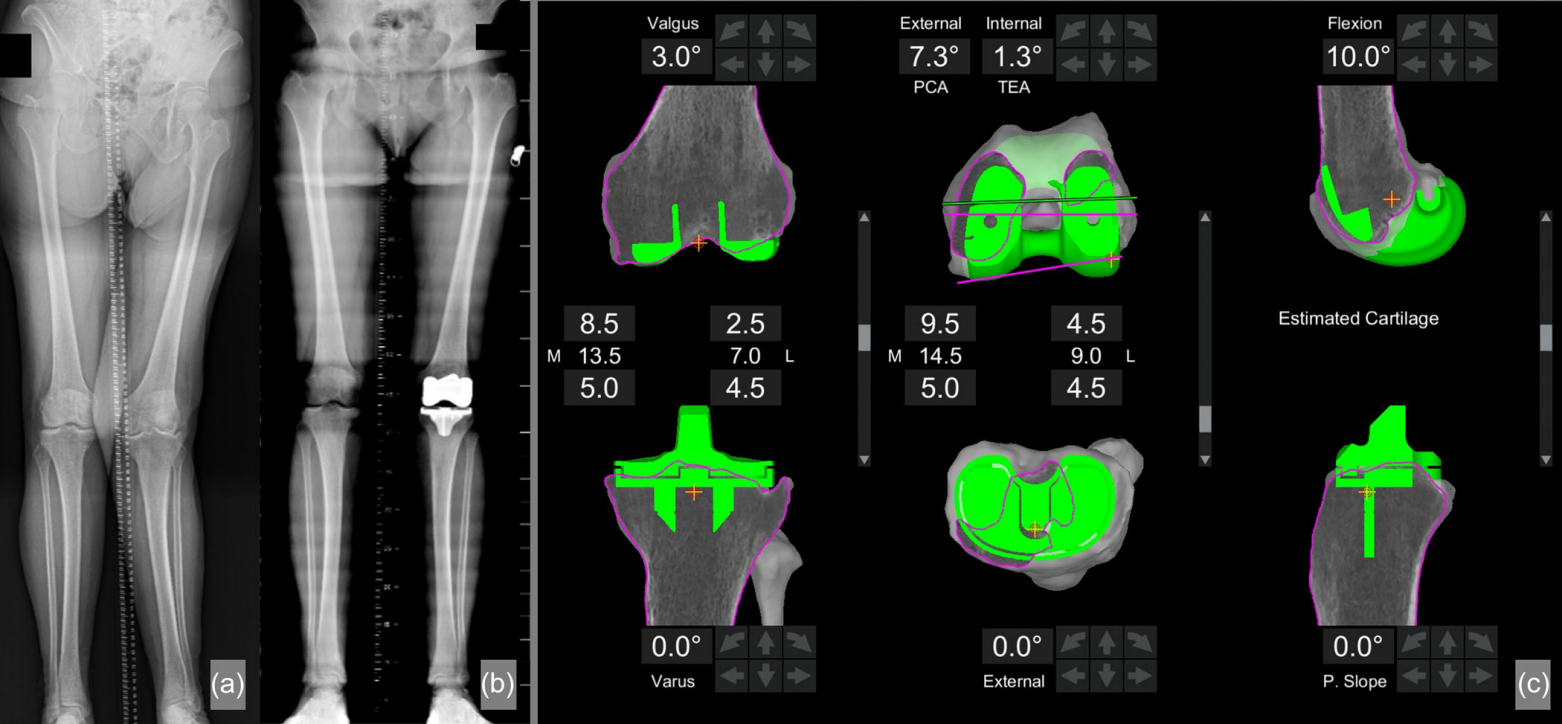
Seventy‐three‐year‐old female patient with a major valgus coronal deformity, presenting a preoperative mechanical hip–knee–ankle (mHKA) angle of 192° on the left side, corrected to 185° after total knee arthroplasty (TKA). (a) Preoperative long‐leg standing radiograph; (b) postoperative long‐leg standing radiograph; (c) intraoperative screenshot of TKA performed with the Mako® robotic system (Stryker®), showing planning adjustments according to functional knee positioning principles.

**Figure 4:**
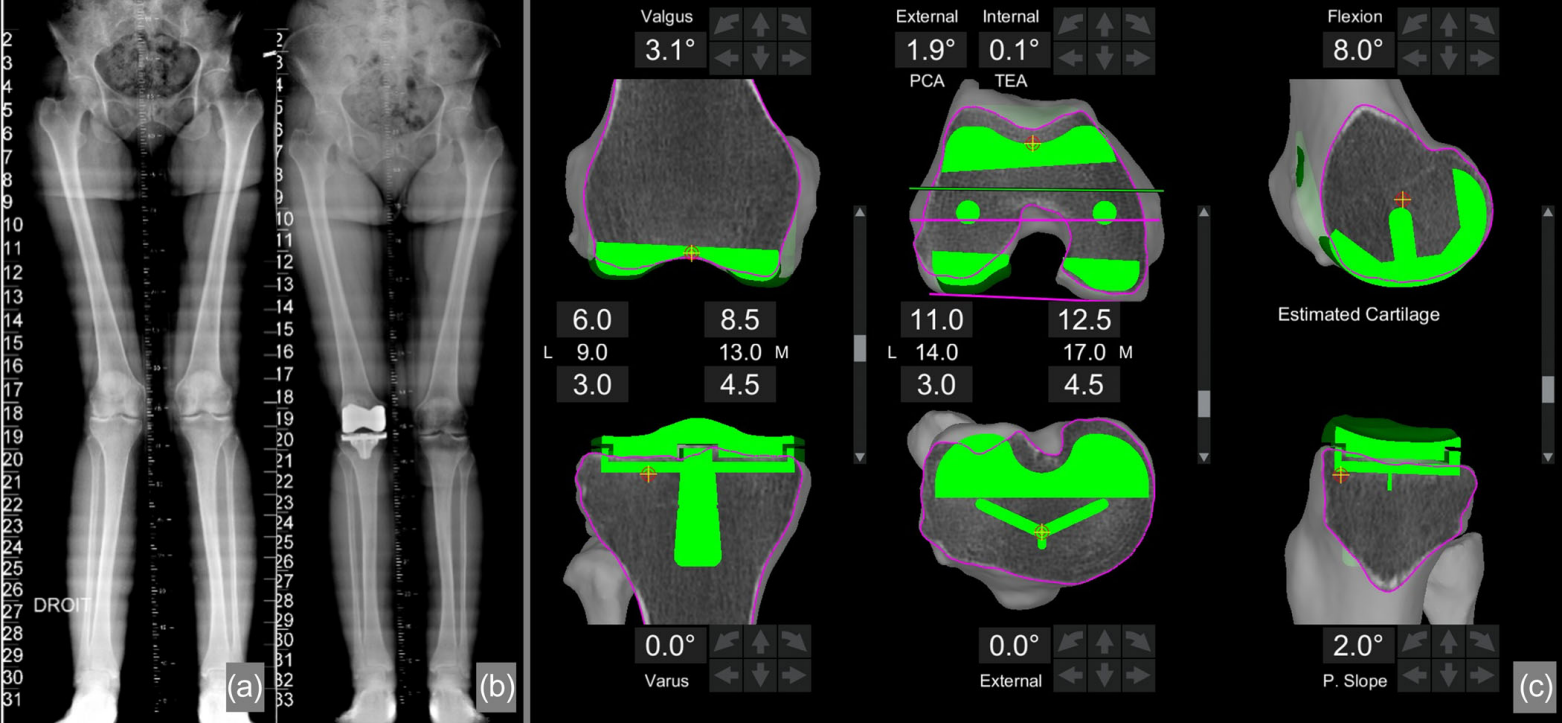
Seventy‐two‐year‐old female patient with a major valgus coronal deformity, presenting a preoperative mechanical hip–knee–ankle (mHKA) angle of 188° on the right side, corrected to 187° after total knee arthroplasty (TKA). (a) Preoperative long‐leg standing radiograph; (b) postoperative long‐leg standing radiograph; (c) intraoperative screenshot of TKA performed with the Mako® robotic system (Stryker ®), showing planning adjustments according to functional knee positioning principles.

All surgeries were carried out using the Mako robotic‐arm‐assisted system (Stryker, Mako Surgical Corp.). Every patient received a Triathlon Total Knee System implant (Stryker, Mako Surgical Corp.), with either a posterior stabilised (PS) or cruciate substituting (CS) liner. Procedures were performed at a single high‐volume centre with expertise in both primary and revision arthroplasty. A standardised surgical approach, following FA or FKP principles, was consistently applied [31, 32] (Figures 1, 2, 3, 4).

Patients were excluded if TKA was performed using mechanical alignment principles because of pre‐existing soft tissue issues, such as prior fractures, periarticular osteotomies, or major ligament injuries requiring deviation from FA. Further exclusions included patients with follow‐up shorter than 24 months and those with incomplete preoperative imaging or missing intraoperative robotic data to maintain consistency in the study methodology.

Demographic information, including age, gender and body mass index (BMI), was collected for all patients. Preoperative assessments included measurements of knee range of motion (ROM) and the knee society score (KSS) for both functional and clinical knee evaluation [16]. Imaging studies consisted of anteroposterior, lateral, Rosenberg, sunrise and full‐length standing X‐rays. From these images, the mHKA, lateral distal femoral angle (LDFA), medial proximal tibial angle (MPTA) and tibial slope (TS) were calculated [15]. Robotic system data provided detailed information on component positioning, using real‐time data obtained from preoperative CT scans. For the femoral component, parameters included flexion/extension relative to the mechanical axis (MA), varus/valgus alignment of the distal femoral cut and rotational alignment based on the surgical transepicondylar axis (sTEA). For the tibial component, varus/valgus alignment of the proximal cut and posterior tibial slope were assessed.

At the final follow‐up, clinical outcomes included KSS‐knee and KSS‐function scores, the Forgotten joint score (FJS‐12) and the Kujala anterior knee pain scale (AKPS), along with ROM measures for recurvatum, flexion contracture and maximum flexion [16, 24]. Data on septic and aseptic complications, reoperations and revision surgeries were also collected. A survival analysis of the implants was conducted, evaluating both all‐cause and aseptic revision rates between the study groups.

To ensure comparability between groups and minimise selection bias, a 1:1 nearest‐neighbour matching using a caliper of 0.5 units for continuous variables (age, BMI and follow‐up period). A total of 44 patients with major coronal plane deformity were included in the study group and matched with 44 patients from the control group based on similar demographic and preoperative characteristics. The groups, within the two respective analysed parameters, were compared in terms of clinical outcomes, radiographic measurements, robotic data and complication rates.

### Ethical approval

This study was conducted in accordance with the ethical principles outlined in the 1964 Declaration of Helsinki and adhered to HIPAA regulations. Data collection and analysis were performed following the MR004 Reference Methodology established by the French Commission Nationale de l'Informatique et des Libertés (Ref. 2229975V0). Informed consent was obtained from all participants.

### Statistical analysis

Continuous variables were reported as mean values with standard deviations (SD), while categorical variables were described using frequencies and percentages. The Shapiro–Wilk test was applied to assess the normality of data distribution. Depending on data distribution, comparisons between groups were performed using either the *t*‐test or the Mann–Whitney *U* test. Categorical variables were compared using the chi‐square test. Implant survival was examined to assess differences in revision rates between groups. A 95% confidence interval was used, and statistical significance was set at a *p*‐value < 0.05. All radiographic measurements were independently performed by two investigators, showing excellent interobserver reliability with intraclass correlation coefficients (ICCs) of 0.90 (95% CI: 0.86–0.94) for preoperative mHKA, 0.91 (95% CI: 0.87–0.95) for postoperative mHKA, 0.89 (95% CI: 0.84–0.93) for preoperative LDFA, 0.91 (95% CI: 0.87–0.94) for postoperative LDFA, 0.90 (95% CI: 0.85–0.94) for preoperative MPTA, 0.92 (95% CI: 0.88–0.95) for postoperative MPTA, and 0.89 (95% CI: 0.84–0.93) for posterior tibial slope. To define the minimal clinically important difference (MCID), the distribution‐based SD method was used, considering the MCID as equivalent to 0.5 × SD. All statistical analyses were conducted using Python version 3.11 (Python Software Foundation) and the statsmodels library (v0.13).

## RESULTS

At the time of the final follow‐up, 88 patients were assessed, with a mean follow‐up duration of 2.8 ± 0.9 years. The cohort comprised 56 women (63.6%), with an average age of 68.8 ± 10.1 years and a mean BMI of 27.1 ± 4.7 kg/m². A CS liner was used in 31 cases (35.3%), while a PS liner was chosen in 57 patients (64.8%).

In the study group, 23 patients (52.3%) presented with major varus deformity, with a mean mHKA of 163.6 ± 2.5°, while 21 patients (47.7%) had major valgus deformity, with a mean mHKA of 192.0 ± 2.7°.

During the preoperative clinical evaluation, the mean KSS‐knee score was 63.9 ± 12.6, and the KSS‐function score averaged 63.2 ± 16.6. Regarding ROM, the mean recurvatum was 0.7 ± 2.1°, the mean flexion contracture was 2.0 ± 3.9°, and the mean maximum flexion reached 118.3 ± 15.3°.

In the preoperative radiographic assessment, the mean mHKA was 178.1 ± 10.4°, the LDFA was 90.5 ± 4.3°, the MPTA was 87.4 ± 4.4°, and the TS was 7.4 ± 3.3°.

Table 1 presents the comparison between the study group and the control group in terms of general and demographic characteristics, as well as preoperative clinical and radiographic data. Statistically significant differences were observed in LDFA (*p* < 0.001) and MPTA (*p* = 0.020).

**Table 1:** Comparison of demographic, preoperative clinical and radiographic data between the group with major coronal plane deformities (defined as ≥15° varus or ≥10° valgus) and the control group.

	Study group (*N* = 44)	Control group (*N* = 44)	*p* value
**Demographic and general data**			
Sex (female)	65.9% (29)	61.4% (27)	0.83
Age (years)	70.5 ± 7.9	69.1 ± 11.5	0.11
BMI (kg/m²)	26.3 ± 4.4	27.8 ± 4.9	0.14
CS liner	40.1% (18)	29.5% (13)	0.38
Follow‐up (years)	2.9 ± 0.8	2.9 ± 0.8	0.42
**Preoperative data**			
KSS knee	62.8 ± 13.3	64.9 ± 11.7	0.45
KSS function	63.2 ± 14.2	64.0 ± 18.6	0.84
Recurvatum (degrees)	0.9 ± 2.5	0.6 ± 1.6	0.45
Flexion contracture (degrees)	1.6 ± 3.5	2.4 ± 4.3	0.36
Maximum flexion (degrees)	116.9 ± 19.0	119.7 ± 11.1	0.40
mHKA (degrees)	177.1 ± 14.5	179.1 ± 3.4	0.37
LDFA (degrees)	88.8 ± 5.2	92.1 ± 2.5	<0.001
MPTA (degrees)	86.4 ± 5.9	88.5 ± 1.6	0.02
TS (degrees)	7.5 ± 3.0	7.3 ± 3.4	0.82

Abbreviations: BMI, body mass index; CS, cruciate‐substituting; KSS, knee society score; LDFA, lateral distal femoral angle; mHKA, mechanical hip–knee–ankle angle, MPTA, medial proximal tibial angle; TS, tibial slope.

The Mako data on tibial component positioning showed an average varus alignment of 2.5 ± 2.0° and an average slope of 0.7 ± 1.0°. For the femoral component, the mean valgus was 0.8 ± 1.9°, the average implant flexion was 7.5 ± 2.3°, and the mean external rotation was 0.2 ± 1.6°.

Table 2 presents data on implant positioning, following the principles of FA, also referred to as FKP. The only statistically significant difference was observed in the coronal plane alignment of the femoral component, which showed reduced valgus in the study group (0.2 ± 2.2° vs. 1.5 ± 1.7°, *p* = 0.002).

**Table 2:** Comparison of robotic implant positioning data, according to the principles of functional alignment, between the group with major coronal plane deformities (defined as ≥15° varus or ≥10° valgus) and the control group.

Mako data on implant positioning			
Values (degrees)	Study group (*N* = 44)	Control group (*N* = 44)	*p* value
Femoral valgus	0.2 ± 2.2	1.5 ± 1.7	0.002
Femoral flexion	7.4 ± 2.2	7.5 ± 2.6	0.92
Femoral external rotation	0.3 ± 1.6	0.1 ± 1.7	0.51
Tibial varus	2.7 ± 2.3	2.4 ± 1.5	0.39
Tibial slope	0.8 ± 1.0	0.7 ± 0.77	0.61

At the final follow‐up, patients in the study group reported a significantly higher FJS‐12 score compared to the control group (83.9 ± 20.2 vs. 74.9 ± 19.0; *p* = 0.04), indicating better joint perception. The overall FJS‐12 score was 79.7 ± 19.8, with a MCID of 9.9. Therefore, the difference of 9.0 points between the two groups does not reach clinical significance, as it falls below the MCID threshold. Radiographically, a significant difference in postoperative limb alignment was observed, with a less neutral mHKA in the study group (176.8 ± 4.7° vs. 180.0 ± 3.0°; *p* = 0.002). No other differences were observed. Table 3 reports the clinical and radiographic comparison between the two groups at the final follow‐up.

**Table 3:** Comparison of clinical and radiographic outcomes at final follow‐up between the group with major coronal plane deformities (defined as ≥15° varus or ≥10° valgus) and the control group.

Postoperative data			
	Study group (*N* = 44)	Control group (*N* = 44)	*p* value
KSS knee	91.2 ± 16.0	91.0 ± 9.3	0.93
KSS function	90.7 ± 12.1	88.8 ± 10.5	0.42
Recurvatum (degrees)	0.6 ± 1.7	0.9 ± 1.8	0.53
Flexion contracture (degrees)	0.1 ± 0.8	0.2 ± 1.5	0.69
Maximum flexion (degrees)	126.0 ± 11.8	125.7 ± 11.0	0.89
FJS‐12	83.9 ± 20.2	74.9 ± 19.0	0.04
AKPS	92.4 ± 13.6	88.2 ± 11.7	0.22
mHKA (degrees)	176.8 ± 4.7	180.0 ± 3.0	0.002
LDFA (degrees)	90.6 ± 2.8	91.3 ± 2.4	0.28
MPTA (degrees)	88.6 ± 2.7	88.7 ± 2.2	0.89

Abbreviations: AKPS, anterior knee pain scale; FJS‐12, forgotten joint score; KSS, knee society score; LDFA, lateral distal femoral angle; mHKA, mechanical hip–knee–ankle angle; MPTA, medial proximal tibial angle.

Four patients (4.5%) required manipulation under anaesthesia (MUA) or arthroscopic arthrolysis within 6 months postoperatively due to persistent stiffness. One patient (1.1%) underwent DAIR (debridement, antibiotics and implant retention) for an acute periprosthetic joint infection, while another (1.1%) required revision of the tibial component. The final implant survival rate was 97.7%, with only 2.3% of patients requiring partial or complete revision. No statistically significant differences were observed in terms of reoperation rate (9.1% vs. 4.5%, *p* = 0.668) or mechanical failure (2.3% vs. 0%, *p* = 0.987).

## DISCUSSION

This study shows that robotic‐assisted TKA performed with FA principles can be applied reliably in patients with major coronal plane deformities, providing complication and revision rates comparable to those observed in patients with less severe deformities. These results are clinically relevant, since managing knees with large varus or valgus deviations has traditionally been considered more challenging, with concerns regarding residual imbalance, suboptimal implant positioning and higher risk of failure [6, 25]. By demonstrating no increase in adverse events, the findings support the safety of functional positioning even in complex anatomical situations and add evidence to the growing body of literature supporting robotic technologies in TKA [12, 22, 27, 35].

An important observation of this study concerns the PROMs. The FJS‐12 was significantly higher in patients with severe deformities compared to the control group, although the absolute difference did not exceed the MCID threshold. This suggests that while the magnitude of difference may not be sufficient to claim clear superiority in clinical terms, the subjective perception of improvement was greater in the study group. One possible explanation is that patients with major deformities start from worse functional and symptomatic conditions, as only partially captured by preoperative KSS scores, and therefore perceive the postoperative result more positively. This interpretation highlights the importance of considering baseline status when comparing PROMs across different patient subgroups and may reflect the fact that statistical significance does not always correspond to clinical relevance [30].

Recent evidence has shown that robotic‐assisted TKA can facilitate intra‐articular correction of severe extra‐articular deformities while reducing the need for extensive soft tissue release [10, 11, 36]. Although using an imageless robotic system, in a prospective series of 14 patients with femoral or tibial malunions, robotic TKA using a FA strategy achieved significant improvements in mechanical alignment and clinical scores, without major complications or the use of constrained implants. Notably, Oxford knee score (OKS) and KSS improved substantially at short‐term follow‐up [33].

Recent evidence has highlighted the impact of preoperative coronal alignment on intraoperative adjustments in robotic‐assisted image‐based TKA performed under FA principles. In a retrospective series of 355 knees, varus deformities required greater tibial varus positioning, whereas valgus deformities necessitated increased femoral valgus positioning and specific modifications in bone resections. Despite these distinct intraoperative strategies, postoperative functional outcomes, including KSS, FJS and AKPS, were comparable between varus and valgus knees, with implant survivorship exceeding 98% in both groups. These findings support the adaptability of FA in robotic TKA, demonstrating that individualised implant positioning can accommodate major deformities without compromising short‐term outcomes or implant longevity [20, 33].

Radiographic analysis also provides interesting insights. The postoperative mHKA was less neutral in the study group compared to controls (176.8 ± 4.7° vs. 180.0 ± 3.0°; *p* = 0.002), reflecting different correction patterns between groups. In particular, valgus deformities tended to be corrected toward neutral, while varus deformities were more often left in slight residual varus, generating outliers within the control cohort. These findings align with the principles of FA, which aim to balance the knee within an anatomically acceptable range rather than forcing all cases into strict mechanical neutrality [17, 31, 32].

Importantly, despite the presence of residual alignment variability, no negative impact on implant survival or complication rates was observed. This reinforces the concept that patient‐specific alignment strategies, facilitated by robotic assistance, may provide stable results without compromising safety and without an increase in early complications or revision rates, even in cases of extra‐articular or intra‐articular deformity, as demonstrated in other studies [5, 9, 33].

From a patient perspective, individuals with severe deformities may perceive a greater improvement in joint function, even if differences in PROMs do not exceed the thresholds for clinical significance [21]. From a radiographic perspective, the findings emphasise that residual varus in selected cases does not necessarily compromise clinical outcomes when the joint is balanced according to FA principles [34, 37].

This study has several strengths, including the use of a prospectively maintained database, standardised surgical techniques in a high‐volume centre, and robust radiographic and clinical follow‐up. One of the strengths of this study is one of the few studies to report both clinical outcomes and implant failure rates for total knee arthroplasties performed with an image‐based robotic system following FA principles in the presence of major lower limb deformities (≥15° varus or ≥10° valgus).

Limitations include its retrospective comparative design, the relatively short follow‐up of less than 3 years, and the limited sample size, which may reduce the ability to detect differences in rare complications or long‐term survival. Furthermore, only one validated PROM (FJS‐12) reached statistical significance, and the lack of additional quality‐of‐life measures may restrict the interpretation of subjective improvements. Future studies with larger cohorts and longer follow‐up will be important to determine whether the trends observed here persist over time and translate into clinically relevant differences in survivorship and patient satisfaction.

## CONCLUSIONS

Robotic‐assisted TKA performed with FA/FKP principles appears to be a feasible option for patients with major coronal plane deformities, achieving short‐term complication and revision rates comparable to those observed in patients with preoperative neutral alignment. While patients with severe deformities reported higher FJS‐12 scores, this difference did not exceed the MCID threshold and should therefore be interpreted with caution. The radiographic differences observed in postoperative alignment suggest that respecting anatomical individuality rather than enforcing mechanical neutrality may be appropriate and does not increase early complication risks. These findings support the application of FA/FKP in complex primary TKA as a viable and reproducible strategy.

## AUTHOR CONTRIBUTIONS

**Luca Andriollo**: Conceptualisation; methodology; data curation; writing original draft. **Emanuele Diquattro**: Conceptualisation; methodology; data curation; writing original draft. **Christos Koutserimpas**: Data curation and review; editing original draft. **Giovan Giuseppe Mazzella**: Data curation and review; editing original draft. **Giulio Bonat**: Data inspection. **Elvire Servien**: Supervision; reviewing and editing. **Cécile Batailler**: Supervision; reviewing and editing. **Sébastien Lustig**: Conceptualisation; supervision; validation; reviewing; editing.

## CONFLICT OF INTEREST STATEMENT

Christos Koutserimpas is consultant for Smith and Nephew. Cécile Batailler and Elvire Servien are consultant for Stryker. Sébastien Lustig: Consultant for Stryker, Smith and Nephew, Heraeus, Depuy Synthes. Institutional research support to Lepine and Amplitude. Editorial Board for Journal of Bone and Joint Surgery. The remaining authors declare no conflicts of interest.

## ETHICS STATEMENT

All procedures were performed in accordance with the ethical standards of the institutional and/or national research committee, the 1964 Helsinki Declaration and its later amendments or comparable ethical standards. Data collection and analysis were carried out in accordance with MR004 Reference Methodology from the Commission Nationale de l'Informatique et des Libertés (Ref. 2226075) obtained on April 19, 2022. The study was registered and filed on the Health Data Hub website. Written informed consent was obtained from all patients and/or families.

## Data Availability

The datasets used and analysed during the current study are available from the corresponding author on reasonable request, subject to approval by the ethics committees of Croix‐Rousse Hospital.

## References

[1] Andriollo L , Gregori P , Koutserimpas C , Servien E , Batailler C , Lustig S . Beyond the coronal plane in robotic total knee arthroplasty‐Part 2: combined flexion does not affect outcomes. Knee Surg Sports Traumatol Arthrosc. 2025;33(8):2939–2949. 10.1002/ksa.12660 PMC12310087 40145260

[2] Andriollo L , Koutserimpas C , Gregori P , Servien E , Batailler C , Lustig S . Beyond the coronal plane in robotic total knee arthroplasty‐Part 1: variations in tibial slope and distal femoral flexion do not affect outcomes. Knee Surg Sports Traumatol Arthrosc. 2025;33(8):2928–2938. 10.1002/ksa.12658 PMC12310085 40130477

[3] Andriollo L , Koutserimpas C , Gregori P , Servien E , Batailler C , Lustig S . A new parameter in the era of robotic total knee arthroplasty: coronal alignment at 90° of flexion impacts clinical outcomes. Knee Surg Sports Traumatol Arthrosc. 2025;33(7):2581–2591. 10.1002/ksa.12648 PMC12205417 40099499

[4] Andriollo L , Picchi A , Demattia G , Marescalchi M , Sangaletti R , Benazzo F , et al. Imageless robotic surgery and a personalized approach: optimizing TKA after ACL reconstruction. Knee. 2025;57:353–360. 10.1016/j.knee.2025.09.007 41067207

[5] Batailler C , Lording T , Libert T , Servien E , Lustig S . Achieving better clinical outcomes after total knee arthroplasty in knees with valgus deformity: the role of alignment strategies. J Bone Jt Surg. 2025;107(2):152–162. 10.2106/JBJS.24.00207 39591439

[6] Beckers G , Kiss M‐O , Massé V , Malavolta M , Vendittoli P‐A . Personalized total knee arthroplasty in patients with extra‐articular deformities. EFORT Open Rev. 2024;9(7):646–657. 10.1530/EOR-23-0215 PMC11297404 38949174

[7] Cholewinski P , Putman S , Vasseur L , Migaud H , Duhamel A , Behal H , et al. Long‐term outcomes of primary constrained condylar knee arthroplasty. Orthop Traumatol Surg Res. 2015;101(4):449–454. 10.1016/j.otsr.2015.01.020 25952710

[8] Deckey DG , Rosenow CS , Verhey JT , Brinkman JC , Mayfield CK , Clarke HD , et al. Robotic‐assisted total knee arthroplasty improves accuracy and precision compared to conventional techniques. Bone Jt J. 2021;103–B(6 Supple A):74–80. 10.1302/0301-620X.103B6.BJJ-2020-2003.R1 34053292

[9] Eu WC , Yuik Ho JP , Kunalan G . Functional alignment is a feasible alignment strategy in robotic assisted total knee arthroplasty for knee osteoarthritis with extra‐articular deformity ‐ a case series. SICOT‐J. 2025;11:2. 10.1051/sicotj/2024059 PMC11727079 39803978

[10] Graichen H , Avram GM , Strauch M , Kaufmann V , Hirschmann MT . Tibia‐first, gap‐balanced patient‐specific alignment restores bony phenotypes and joint line obliquity in a great majority of varus and straight knees and normalises valgus and severe varus deformities. Knee Surg Sports Traumatol Arthrosc. 2024;32(5):1287–1297. 10.1002/ksa.12145 38504509

[11] Graichen H , Avram GM , Zambianchi F , Graichen NM , Catani F , Lustig S , et al. Bony alignment decisions affect patient‐specific laxity phenotype patterns significantly, independent of the deformity. Knee Surg Sports Traumatol Arthrosc. 2025;33(10):3637–3645. 10.1002/ksa.12730 40583413

[12] Gregori P , Koutserimpas C , Giovanoulis V , Batailler C , Servien E , Lustig S . Functional alignment in robotic‐assisted total knee arthroplasty for valgus deformity achieves safe coronal alignment and excellent short‐term outcomes. Knee Surg Sports Traumatol Arthrosc. 2025;33(6):2187–2196. 10.1002/ksa.12585 PMC12104782 39821487

[13] Hess S , Chelli S , Leclercq V , Lustig S , Graichen H , Hirschmann MT . Three‐compartment phenotype concept of total knee arthroplasty alignment: mismatch between distal femoral, posterior femoral, and tibial joint lines. J Arthroplasty. 2025;40(8):2023–2034. 10.1016/j.arth.2025.02.015 40049560

[14] Hirschmann MT , Avram G , Graichen H , Tandogan RN , Mengis N , Zaffagnini S . Same same but different—image‐based versus imageless robotic‐assisted total knee arthroplasty! J Exp Orthop. 2024;11(4):e70062. 10.1002/jeo2.70062 PMC11489859 39429890

[15] Hiyama S , Rao RP , Takahashi T , Palan J , Pandit H . Biplanar radiographic analysis of knee alignment: a stepwise approach for phenotype classification and knee arthroplasty planning. EFORT Open Rev. 2025;10(10):745–755. 10.1530/EOR-2024-0155 PMC12494059 41031620

[16] Insall JN , Dorr LD , Scott RD , Scott WN . Rationale of the knee society clinical rating system. Clin Orthop Relat Res. 1989;(248):13–14. 2805470

[17] Kafelov M , Batailler C , Shatrov J , Al‐Jufaili J , Farhat J , Servien E , et al. Functional positioning principles for image‐based robotic‐assisted TKA achieved a higher Forgotten Joint Score at 1 year compared to conventional TKA with restricted kinematic alignment. Knee Surg Sports Traumatol Arthrosc. 2023;31(12):5591–5602. 10.1007/s00167-023-07609-3 37851026

[18] Koutserimpas C , Andriollo L , Gregori P , Servien E , Batailler C , Lustig S . Robotic total knee arthroplasty with functional alignment yields comparable outcomes across age and gender groups. J ISAKOS. 2025;14:100930. 10.1016/j.jisako.2025.100930 40716713

[19] Koutserimpas C , Andriollo L , Gregori P , Zambianchi F , Tsiridis E , Catani F , et al. Revisiting terminology: the transition from “functional alignment” to “functional knee positioning. Knee Surg Sports Traumatol Arthrosc. 2025;33(6):1948–1953. 10.1002/ksa.12667 40167115

[20] Koutserimpas C , Caria C , Gregori P , Andriollo L , Servien E , Batailler C , et al. Functional alignment in robotic total knee arthroplasty achieves comparable outcomes in varus and valgus knees despite distinct intraoperative strategies: analysis of 355 consecutive cases. Knee Surg Sports Traumatol Arthrosc. 2025;33(11):3925–3934. 10.1002/ksa.12764 PMC12582244 40621947

[21] Koutserimpas C , Garibaldi R , Olivier F , Servien E , Batailler C , Lustig S . Tibial implant varus >3° does not adversely affect outcomes or revision rates in functionally aligned image‐based robotic total knee arthroplasty in a minimum of 2‐year follow‐up. Knee Surg Sports Traumatol Arthrosc. 2025;33(8):2917–2927. 10.1002/ksa.12659 40130488

[22] Koutserimpas C , Gregori P , Andriollo L , Diquattro E , Servien E , Batailler C , et al. Impact of high body mass index on functionally aligned image‐based robotic total knee arthroplasty: comparable functional outcomes but higher mechanical failures. J ISAKOS. 2025;12:100861. 10.1016/j.jisako.2025.100861 40210164

[23] Marchand R , Sodhi N , Khlopas A , Sultan A , Higuera C , Stearns K , et al. Coronal correction for severe deformity using robotic‐assisted total knee arthroplasty. J Knee Surg. 2017;31:002–005. 10.1055/s-0037-1608840 29179223

[24] Reiter CR , Abraham VM , Riddle DL , Patel NK , Goldman AH . Patient reported outcome measures (PROMs) as primary and secondary outcomes in total hip and knee arthroplasty randomized controlled trials: a systematic review. Arch Orthop Trauma Surg. 2024;144(5):2257–2266. 10.1007/s00402-024-05242-4 38561507

[25] Ritter MA , Davis KE , Davis P , Farris A , Malinzak RA , Berend ME , et al. Preoperative malalignment increases risk of failure after total knee arthroplasty. J Bone Jt Surg Am Vol. 2013;95(2):126–131. 10.2106/JBJS.K.00607 23324959

[26] Röhner E , Benad K , Zippelius T , Kloss N , Jacob B , Kirschberg J , et al. Good clinical and radiological results of total knee arthroplasty using varus valgus constrained or rotating hinge implants in ligamentous laxity. Knee Surg Sports Traumatol Arthrosc. 2019;27(5):1665–1670. 10.1007/s00167-018-5307-6 30456570

[27] Rossi SMP , Sangaletti R , Andriollo L , Matascioli L , Benazzo F . The use of a modern robotic system for the treatment of severe knee deformities. Technol Health Care. 2024;32(5):3737–3746. 10.3233/THC-231261 38251078

[28] Rossi SMP , Sangaletti R , Perticarini L , Terragnoli F , Benazzo F . High accuracy of a new robotically assisted technique for total knee arthroplasty: an in vivo study. Knee Surg Sports Traumatol Arthrosc. 2023;31(3):1153–1161. 10.1007/s00167-021-06800-8 PMC8723813 34981162

[29] Schelker BL , Moret CS , Sava MP , von Eisenhart‐Rothe R , Graichen H , Arnold MP , et al. The impact of different alignment strategies on bone cuts in total knee arthroplasty for varus knee phenotypes. Knee Surg Sports Traumatol Arthrosc. 2023;31(5):1840–1850. 10.1007/s00167-023-07351-w PMC10089997 36811657

[30] Schöner L , Steinbeck V , Busse R , Marques CJ . Satisfied with the worst health outcomes or unsatisfied with the best: explaining the divergence between good patient‐reported outcomes and low satisfaction and vice versa among knee arthroplasty patients ‐ a retrospective cohort study. J Orthop Surg. 2025;20(1):88. 10.1186/s13018-025-05507-7 PMC11755965 39849486

[31] Shatrov J , Battelier C , Sappey‐Marinier E , Gunst S , Servien E , Lustig S . Functional alignment philosophy in total knee arthroplasty ‐ rationale and technique for the varus morphotype using a CT based robotic platform and individualized planning. SICOT‐J. 2022;8:11. 10.1051/sicotj/2022010 PMC8973302 35363136

[32] Shatrov J , Foissey C , Kafelov M , Batailler C , Gunst S , Servien E , et al. Functional alignment philosophy in total knee arthroplasty‐rationale and technique for the valgus morphotype using an image based robotic platform and individualized planning. J Pers Med. 2023;13(2):212. 10.3390/jpm13020212 PMC9961945 36836446

[33] Soundarrajan D , Kumar KM , Singh R , Rajasekaran RB , Palanisami D , Natesan R , et al. Imageless robotic‐assisted total knee arthroplasty allows intra‐articular correction of severe extra‐articular deformities using functional alignment and desired under‐correction. Int Orthop. 2025;49(8):1869–1878. 10.1007/s00264-025-06563-8 40402236

[34] Vermorel P‐H , Ciccullo C , De Berardinis L , Gigante AP , Neri T , Philippot R . Does robotic arm‐assisted total knee arthroplasty have a role to play in large deformities? SICOT‐J. 2024;10:50. 10.1051/sicotj/2024046 PMC11580621 39570039

[35] Xing P , Qu J , Feng S , Guo J , Huang T . Comparison of the efficacy of robot‐assisted total knee arthroplasty in patients with knee osteoarthritis with varying severity deformity. J Orthop Surg. 2024;19(1):872. 10.1186/s13018-024-05372-w PMC11668079 39719605

[36] Yang Y , Jiang L , Zhou X , Zhou X , Chen H , Chen Z . Robotic‐assisted total knee arthroplasty improves implant position and early functional recovery for the knee with severe varus/valgus deformity. BMC Musculoskelet Disord. 2024;25(1):92. 10.1186/s12891-024-07203-9 PMC10809628 38267884

[37] Zhang J , Ndou WS , Ng N , Gaston P , Simpson PM , Macpherson GJ , et al. Robotic‐arm assisted total knee arthroplasty is associated with improved accuracy and patient reported outcomes: a systematic review and meta‐analysis. Knee Surg Sports Traumatol Arthrosc. 2022;30(8):2677–2695. 10.1007/s00167-021-06464-4 PMC9309123 33547914

